# Editorial: Advancements in multi-omics and bioinformatics for the management of solid malignancies

**DOI:** 10.3389/fimmu.2025.1640571

**Published:** 2025-07-07

**Authors:** Noha M. Elemam, Pengpeng Zhang, Wantao Wu, Qiang Wang, Jian Song, Rana A. Youness

**Affiliations:** ^1^ Clinical Sciences Department, College of Medicine, University of Sharjah, Sharjah, United Arab Emirates; ^2^ Research Institute for Medical and Health Sciences, University of Sharjah, Sharjah, United Arab Emirates; ^3^ Department of Thoracic Surgery, The First Affiliated Hospital of Nanjing Medical University, Nanjing, China; ^4^ Department of Thyroid and Breast Surgery, The Second Affiliated Hospital, Chongqing Medical University, Chongqing, China; ^5^ Center for Translational Research in Hematological Malignancies, Houston Methodist Neal Cancer Center/Houston Methodist Research Institute, Houston Methodist Hospital, Houston, TX, United States; ^6^ Institute of Physiological Chemistry and Pathobiochemistry, University of Münster, Münster, Germany; ^7^ Department of Molecular Biology and Biochemistry, Molecular Genetics Research Team (MGRT), Faculty of Biotechnology, German International University (GIU), New Administrative Capital, Cairo, Egypt

**Keywords:** multi-omics integration, precision oncology advancements, bioinformatics in solid tumors, computational tools for cancer analysis, translational multi-omics research

In an era where precision oncology is paramount, the fusion of multi-omics and bioinformatics has emerged as a revolutionary force in the battle against solid malignancies, which constitute a predominant portion of cancer cases globally. In this Research Topic, several studies highlight the development of innovative computational tools to identify molecular drivers of cancer progression and drug resistance, key insights that enable the design of targeted therapies and strategies to overcome treatment resistance. For instance, a study by Zhao et al. evaluated the accuracy of a robotic magnetic navigation system for intraoral osteotomy in mandibular tumor surgery. Using 3D-printed models from patient CT data, researchers compared traditional surgery to robot-assisted osteotomy. The experimental group demonstrated significantly lower positional and angular errors than the control group, confirming improved accuracy. Despite limitations like soft tissue simulation and potential magnetic interference in clinical settings, the findings support the system’s feasibility. Moreover, the study highlights the potential of electromagnetic navigation in enhancing surgical precision while minimizing tissue damage, emphasizing the need for further refinement of hardware, software, and workflow for broader clinical application. Another study developed and validated a hepatocellular carcinoma (HCC) prognosis-related gene signature (HPRGS) to improve survival prediction and treatment guide. Using transcriptome data from multiple HCC cohorts and 101 machine learning algorithms, four key genes (SOCS2, LCAT, ECT2, TMEM106C) were identified. The HPRGS effectively stratified patients into high- and low-risk groups with distinct survival outcomes, treatment responses, and mutation profiles. Low-risk patients showed better responses to immunotherapy, while linifanib was identified as a potential treatment for high-risk patients. A nomogram incorporating HPRGS supports clinical decision-making, highlighting its utility as an independent prognostic tool for personalized HCC management (Zheng et al.). Regarding glioma, this study tackled the poor prognosis of glioma by integrating multi-omics data and using Non-negative Matrix Factorization to identify two metabolic subtypes with distinct clinical and molecular profiles. Key genes were identified through Weighted Gene Correlation Network Analysis (WGCNA), and a prognostic model was built using 101 machine learning methods. Such results provide novel prognostic framework and highlight the metabolic and immune heterogeneity of glioma, offering deeper insights into its biology and potential therapeutic targets (Hu et al.).

Building on recent advances in cancer genomics, this study explored the underexamined role of chromatin regulators (CRs) in lung adenocarcinoma (LUAD) by constructing a chromatin regulator-related signature (CRRS). This signature, developed using a comprehensive 429-combination machine learning framework to predict patient survival, was robustly validated across multiple datasets. The CRRS was also found to modulate the immune microenvironment, with high-risk patients exhibiting distinct pathway activities, mutation profiles, and immune responses. Notably, Trefoil Factor 1 (TFF1), a chromatin regulator, was identified as a key therapeutic target, as its knockdown in LUAD cells significantly inhibited proliferation, induced apoptosis, and suppressed *in vivo* tumor growth, concluding that chromatin regulators hold promise for prognostic modeling and immune modulation in LUAD (Fan et al.).

An intriguing study was conducted by Luo et al. that investigated the impact of exercise on breast cancer progression using a murine model. Mice subjected to 21 days of voluntary running showed reduced tumor size and weight. RNA sequencing revealed significant upregulation of THSD7B and changes in pathways related to cancer, including microRNAs and calcium signaling. Pan-cancer analyses indicated that THSD7B is variably expressed and associated with favorable prognosis in several cancers. Functional studies confirmed its role in inhibiting breast cancer cell proliferation, migration, and invasion. Therefore, exercise could modulate tumor biology and THSD7B may serve as a prognostic biomarker and therapeutic target in cancer management (Luo et al.). Furthermore, another study by Fang et al. aimed at developing a nomogram to predict survival in hormone receptor-positive mucinous breast carcinoma (HR+ MBC) patients and assessed the impact of neoadjuvant chemotherapy (NAC). Using data from 6,927 patients, eight independent prognostic factors were identified. The nomogram accurately stratified risk, with high-risk patients showing poorer survival. Notably, NAC did not improve long-term survival compared to adjuvant chemotherapy, suggesting limited benefit in this group (Fang et al.).

Colon adenocarcinoma (COAD), a highly prevalent and lethal malignancy, has a complex pathogenesis where ubiquitin-mediated regulation of key cellular processes plays a significant role. This study investigated the role of ubiquitination in COAD by integrating transcriptomic, single-cell, and clinical data to develop a prognostic risk signature. Using Cox and LASSO regression, researchers identified ubiquitination-related genes that stratify patients by survival risk, with high-risk scores linked to poorer outcomes and increased immune cell infiltration. Functional assays showed that silencing ASNS, a key gene in the signature, significantly reduced COAD cell activity and migration. Thus, ubiquitination features could be effective prognostic indicators with ASNS as a promising biomarker and potential therapeutic target in COAD (Wang et al.).

On the other hand, another study identified key stem cell-related genes in prostate adenocarcinoma (PRAD) using single-cell analysis and machine learning (Wang et al.). Among 15 crucial genes, HSPE1 emerged as a vital marker associated with PRAD diagnosis, prognosis, and immune infiltration. HSPE1 was identified through random forest analysis and validated by immunofluorescence staining in 60 PRAD tissue samples, confirming its upregulation and correlation with poor patient outcomes. Molecular docking further explored HSPE1’s interactions with therapeutic compounds. These findings highlight HSPE1 as a promising biomarker and therapeutic target, offering new avenues for early detection and personalized treatment strategies in PRAD (Wang et al.). Similar approaches were explored in a study of osteosarcoma (OS), where they investigated the tumor microenvironment (TME) of OS using bulk and single-cell RNA sequencing data. Analysis revealed high transcriptional heterogeneity in OS cells, with cluster 1 showing strong aggressiveness and poor prognosis. A tumor-infiltrating immune cell (TIIC)-based gene signature was developed using 20 machine learning algorithms to predict survival and immunotherapy response. High TIIC scores correlated with lower immune infiltration and worse outcomes. Also, CLK1 was identified as an oncogene that promotes OS cell proliferation and migration. These findings offer new prognostic tools and highlight CLK1 as a potential therapeutic target in OS treatment (Zhang et al.). Another study on OS used bibliometric analysis of Web of Science literature (2014-2023) to map the evolution of OS metabolomics research, identify key contributors, and predict future trends. The analysis of authors, citations, and keywords revealed research clusters and suggests that upcoming work will likely focus on the TME, molecular mechanisms (including autophagy), and targeted therapies/inhibitors (Tu et al.).

Hypoxia is a hallmark of the tumor microenvironment in pancreatic ductal adenocarcinoma (PDAC), contributing to its aggressive behavior, therapeutic resistance, and poor prognosis by promoting immune evasion, metabolic reprogramming, and resistance to cell death mechanisms such as ferroptosis. By integrating multi-omics data, researchers identified a correlation between hypoxia levels, sulfide quinone oxidoreductase (SQOR) expression, and survival outcomes. A deep learning model was developed to predict hypoxia from whole slide images, and experimental models confirmed that SQOR promotes tumor progression by enhancing ferroptosis resistance. Notably, SQOR inhibition increased ferroptosis sensitivity, and combining SQOR inhibitors with ferroptosis inducers had synergistic anti-tumor effects, highlighting a promising strategy for targeted PDAC therapy (Lin et al.). A comprehensive analysis of gastric adenocarcinoma (STAD) was conducted by Yin et al. through integrating large-scale genomic datasets, spatial transcriptomics, and single-cell RNA sequencing to investigate the prognostic significance of lactylation-related gene sets and mitochondrial functions, as well as to delineate the TME and cellular heterogeneity. The research identified distinct molecular subtypes within STAD associated with unique survival outcomes and immune profiles, leading to the development of prognostic models with enhanced predictive capabilities. Furthermore, the analysis revealed that variations in lactylation could influence immune cell infiltration and responsiveness, suggesting potential for tailored immunotherapy (Yin et al.).

Other approaches including assessment of non-coding RNAs was performed in the study by Zhang et al. with a special focus on the role of circular RNA circTAF4B in bladder cancer (BCa) progression. Researchers identified MFN2 as a binding partner of circTAF4B using RNA pull-down and mass spectrometry. Silencing MFN2 enhanced the inhibitory effects of circTAF4B overexpression on BCa cell growth and migration. Additionally, circTAF4B knockdown suppressed tumor progression by upregulating p27 and blocking AKT signaling. Although circTAF4B binds to MFN2 without altering its expression, it influences BCa through distinct regulatory pathways. These findings highlight circTAF4B as a promising biomarker and therapeutic target in BCa, warranting further exploration of its molecular mechanisms and clinical applications (Zhang et al.).

Published review articles in this Research Topic included a review that highlights the immunoproteasome’s pivotal role in enhancing anti-tumor immune responses by promoting antigen processing and presentation via MHC class I molecules. It explored its involvement in immune surveillance, TME modulation, and its emerging potential as a therapeutic target in cancers such as melanoma, lung, colorectal, and breast cancer. The review discussed immunoproteasome inhibitors like ONX 0914, their synergy with checkpoint inhibitors, and strategies to boost immunoproteasome activity. Challenges including toxicity, resistance, and the need for predictive biomarkers are addressed. Overall, targeting the immunoproteasome offers a promising avenue for improving precision and durability of cancer immunotherapy (Shi et al.). Another review by Diab et al. discussed the role of P-glycoprotein (P-gp) in cancer chemoresistance and the potential of natural products to overcome it. P-gp, a drug efflux transporter, contributes to chemotherapeutic failure by reducing intracellular drug accumulation. Long noncoding RNAs (lncRNAs) such as ODRUL, MALAT1, and ANRIL regulate P-gp and are linked to drug resistance. *In silico* molecular docking identified Delphinidin and Asparagoside-f as potent natural P-gp inhibitors capable of reversing multidrug resistance. Such findings highlight the promise of natural compounds in enhancing chemotherapy sensitivity, though further *in vitro* and *in vivo* validation is essential to confirm their therapeutic potential.

Looking at the immunological aspect of tumors, this study assessed the impact of M2 macrophage infiltration on prognosis in serous ovarian cancer (SOC) and explored the use of histopathological imaging features (HIF) to predict M2 levels using deep learning (Zhao et al.). Analysis of TCGA and external patient data revealed high M2 macrophage infiltration as an independent risk factor for poor survival. A ResNet18-based deep multiple instance learning model using the Mean Probability Method effectively predicted M2 infiltration from histological images, achieving an AUC of 0.75. These findings support the integration of image-based AI tools into SOC prognosis and personalized treatment planning by identifying immune microenvironment markers (Zhao et al.). Another study analyzed the immunological mechanisms and hypertension profiles associated with VEGF inhibitors (VEGFi) and VEGF receptor inhibitors (VEGFRi) using the FDA Adverse Event Reporting System (FAERS), clinical data, and preclinical models. Both inhibitors significantly increased the risk of immune-mediated, blood pressure-related adverse events. VEGFRi induced a more rapid onset, greater blood pressure elevation, and higher incidence of immune-related hypertension compared to VEGFi. The study identified key signaling pathways, such as MAPK and nitric oxide dysregulation, involved in these effects. The findings highlight the need for early and continuous blood pressure monitoring in patients treated with these inhibitors to prevent cardiovascular complications (Kuang et al.). A pan-cancer study investigated the potential of necroptosis as a predictive biomarker for immunotherapy responses. The researchers developed a necroptosis-related gene signature, Necroptosis.Sig, using bulk RNA sequencing data and employed multi-omics approaches to identify key pathways and regulators, highlighting HMGB1 as a critical modulator. Functional validation in A549 lung cancer cells demonstrated that HMGB1 knockdown suppressed tumor proliferation and malignancy, supporting the therapeutic relevance of targeting necroptosis. The study concluded that necroptosis and its regulators, like HMGB1, represent promising tools for advancing precision oncology and improving patient outcomes in immunotherapy (Gao et al.).

Another angle of immunotherapy was investigated in the study which addressed the critical challenge of predicting immunotherapy-related adverse reactions (irAEs) in hepatitis B virus-positive hepatocellular carcinoma (HBV-HCC) patients treated with immune checkpoint inhibitors (ICIs). Analyzing data from 274 patients, researchers developed machine learning models, with the Random Forest model showing the best predictive accuracy. Antiviral therapy and HBV DNA levels were key factors, with antiviral treatment linked to a lower risk of irAEs, possibly through B cell modulation. The findings suggest that antiviral therapy may reduce irAE severity, even without complete viral suppression, offering a potential strategy to improve the safety of immunotherapy in HBV-HCC patients (Pan et al.).

